# Long noncoding RNA *MEG3* inhibits oral squamous cell carcinoma progression via GATA3

**DOI:** 10.1002/2211-5463.13532

**Published:** 2022-12-18

**Authors:** Yan Hu, Feifei Lv, Na Li, Xuewei Yuan, Liru Zhang, Shuangling Zhao, Linyu Jin, Yongle Qiu

**Affiliations:** ^1^ Department of Stomatology Affiliated Hospital of Hebei University Baoding China; ^2^ Department of Stomatology Second Hospital of Shijiazhuang China; ^3^ Department of Stomatology First Outpatient Department of Hebei Province Shijiazhuang China; ^4^ Department of Stomatology, Fourth Affiliated Hospital Hebei Medical University Shijiazhuang China

**Keywords:** GATA3, H3K27me3, m6A, *MEG3*, noncoding RNA, oral squamous cell carcinoma

## Abstract

Oral squamous cell carcinoma (OSCC) accounts for about 90% of oral cancers. Expression of the long noncoding RNA (lncRNA) maternally expressed 3 (*MEG3*) has previously been reported to be downregulated in OSCC, and its overexpression can inhibit proliferation, migration, and invasion and promote apoptosis of OSCC cells. However, the mechanism underlying *MEG3* downregulation in OSCC has not been well characterized. Here we report that low expression of *MEG3* is caused by H3K27me3 modification of the *MEG3* gene locus, and this is associated with the poor prognosis of OSCC. Overexpression of *MEG3* inhibited the proliferation and invasion of OSCC cells. We observed that *MEG3* was modified by m6A and bound to YTHDC1. Enhancer‐controlled genes positively regulated by *MEG3* were functionally enriched for the ‘negative regulation of Wnt signaling pathway’ term, as determined using metascape. GATA3 was predicted to be a transcription factor for these genes, and was demonstrated to bind to *MEG3*. Knockdown of GATA3 countered the effects on proliferation, invasion, and increased transcription of HIC1 and PRICKLE1 induced by *MEG3* overexpression. In conclusion, our data suggest that *MEG3* is downregulated in OSCC due to trimethylation of H3K27 at the *MEG3* gene locus. The inhibitory effect of *MEG3* on proliferation and invasion of OSCC cells was dependent on the binding of GATA3.

## Graphical abstract

We report that the lncRNA maternally expressed 3 (*MEG3*) was downregulated in oral squamous cell carcinoma (OSCC), and this was mediated by H3K27me3 modification of the *MEG3* gene locus. Overexpression of *MEG3* inhibited the proliferation and invasion of OSCC cells. Furthermore, the anticancer function of *MEG3* in OSCC is dependent on the interaction with GATA3.

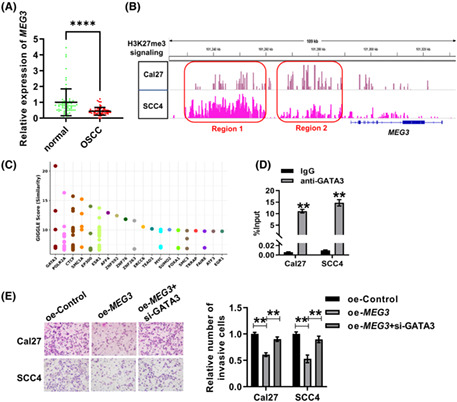

**Abbreviations**
ANOVA: analysis of variance
ATCC: American Type Culture Collection
BRD4: bromodomain containing 4
CCK8: cell counting kit 8
ChIP: chromatin immunoprecipitation
EZH2: enhancer of zeste homolog 2
GATA3: GATA binding protein 3
GEO: Gene Expression Omnibus
H3K27me3: trimethylation of Lys‐27 in histone 3
HIC1: HIC ZBTB transcriptional repressor 1
lncRNA: long noncoding RNA
m6A: N6‐methyladenosine
*MEG3*
: long noncoding RNA maternally expressed 3
NOK: normal oral keratinocyte line
OD: optical density
oe‐Control: empty plasmid
oe‐*MEG3*
: *MEG3* overexpression plasmid
OS: overall survival
OSCC: oral squamous cell carcinoma
PFS: progression‐free survival
PRICKLE1: prickle planar cell polarity protein 1
qRT‐PCR: quantitative real time‐polymerase chain reaction
RIP: RNA immunoprecipitation
si‐GATA3: siRNA targeting GATA3
si‐NC: siRNA negative control
SRAMP: sequence‐based RNA adenosine methylation site predictor
TCGA: The Cancer Genome Atlas
TRF2: telomeric repeat binding factor 2
YTHDC1: YTH domain containing 1

Oral cancer is the sixth most common malignancy worldwide, with about 354 864 new cases and 177 384 deaths annually [1, 2]. As the most common pathological type of oral cancer, oral squamous cell carcinoma (OSCC) accounts for about 90% of oral cancers [3]. The preferred position of OSCC is the tongue and mouth floor. Early symptoms of OSCC are atypical, and most patients are diagnosed at an advanced stage of the disease [4]. Patients with advanced OSCC are prone to lymph node metastasis and recurrence [5]. It is worth noting that when patients present with metastasis outside the anterior lymph nodes, they often develop metastasis to other distant organs [5]. Patients with advanced OSCC have a poor prognosis, with a 5‐year survival of only about 30% [6, 7]. The identification of biomarkers for the diagnosis and treatment of OSCC has great clinical importance.

LncRNA maternally expressed 3 (*MEG3*) is ~ 1600 nucleotides in length [8]. *MEG3* expression is downregulated in a variety of tumors such as gastric cancer, nasopharyngeal carcinoma, colorectal cancer, breast cancer, and prostate cancer [9, 10, 11, 12, 13]. In previous studies, *MEG3* expression was reported to be downregulated in OSCC tissues [14, 15, 16]. Overexpression of *MEG3* could inhibit proliferation, migration, invasion, and promote apoptosis of OSCC cells [14, 15, 16]. However, the mechanism underlying *MEG3* downregulation in OSCC has not been well characterized. Hypermethylation of the gene locus is an essential mechanism for suppressing gene expression [17]. Several studies reveal that the low expression of *MEG3* in adenoma, meningioma, and neuroblastoma is directly related to the trimethylation of Lys‐27 in histone 3 (H3K27me3) of the *MEG3* gene promoter [18, 19, 20]. However, it is unknown whether the downregulation of *MEG3* expression in OSCC is related to H3K27me3 modification of the *MEG3* gene locus.

Recently, the mechanism of *MEG3* inhibiting tumor progression has attracted extensive attention. For example, *MEG3* inhibits hepatocarcinogenesis by inhibiting the activity of telomerase and telomeric repeat binding factor 2 (TRF2) [21]. Chen et al. reported that *MEG3* inhibits multiple myeloma progression through upregulating p53 [22]. *MEG3* inhibits migration and promotes apoptosis of OSCC cells by regulating the JAK/STAT signaling pathway through adsorption of miR‐548d‐3p [14]. Another study has shown that *MEG3* inhibits proliferation and migration of OSCC cells by targeting miR‐21 [15]. In addition, *MEG3* could inhibit proliferation and metastasis of OSCC cells by suppressing the Wnt/β‐catenin signaling pathway [23]. LncRNAs regulate gene expression at multiple levels, including epigenetic, transcriptional, and posttranscriptional levels. *MEG3* is a chromatin‐interacting lncRNA, and its mechanism of regulation of gene expression remains elusive [24]. The detailed mechanism by which *MEG3* inhibits OSCC progression is a subject deserving in‐depth study.

N6‐methyladenosine (m6A) is a prevalent epigenetic modification of RNA [25]. m6A modification is dynamically regulated by methyltransferases (writers), demethylases (erasers), and methyl recognition protein (readers) [26]. m6A modification of lncRNAs influences cancer progression by regulating biological functions associated with cancer [25]. However, whether *MEG3* is modified by m6A in OSCC cells, and whether m6A modification of *MEG3* is associated with *MEG3*‐mediated regulation of the target gene, remains to be elucidated.

The purpose of this study was to elucidate whether the downregulation of *MEG3* in OSCC cells is associated with H3K27me3 at *MEG3* gene locus, and to uncover the transcription factors involved in the cancer‐suppressive function of *MEG3*.

## Materials and methods

### Datasets

Gene expression profiles of 324 OSCC tissues and 32 normal control tissues were downloaded from The Cancer Genome Atlas (TCGA, https://tcga‐data.nci.nih.gov/tcga/) using the r package ‘TCGAbiolinks.’ H3K27me3 ChIP‐seq data of Cal27 and SCC4 cells, as well as H3K27ac ChIP‐seq data of Cal27 cells were downloaded from GSE149670 dataset of the Gene Expression Omnibus (GEO, http://www.ncbi.nlm.nih.gov/geo/).

### Prediction of m6A site of *MEG3*

Sequence‐based RNA adenosine methylation site predictor (SRAMP, http://www.cuilab.cn/sramp) is a database based on a random forest machine‐learning framework, which is able to predict potential m6A sites and calculate the confidence for each m6A site [27]. In this study, potential m6A sites for *MEG3* were predicted by the SRAMP database.

### Identification of H3K27me3 peak, H3K27ac peak and enhancer‐controlled gene

H3K27me3 and H3K27ac peaks were visualized using the integrative genomics viewer (https://igv.org). Enhancers were identified using the homer ‘findPeaks’ package based on H3K27ac ChIP‐seq data of Cal27 cells from GSE149670. The enhancer‐controlled gene was defined as the gene that is nearest to the enhancer region, and was identified by the homer ‘annotatePeaks’ package.

### Gene expression correlation analysis

Pearson's correlation was analyzed for gene expression correlation using r package ‘stats’ based on the data downloaded from TCGA database. *P* < 0.05 and ¦cor¦ ≥ 0.3 were set as the threshold value to screen for genes significantly correlated with *MEG3* expression.

### Biological function analysis

metascape (https://metascape.org/) is an online platform for gene function annotation [28]. Biological function of genes was enriched using metascape in this study.

### Transcription factor prediction

The cistrome data Browser is a website for *cis*‐regulatory information, such as transcription factor binding sites, histone posttranslational modifications, and chromatin endonuclease action sites, of human and mouse [29]. In this study, transcription factors were predicted by the Toolkit for cistrome data Browser (http://dbtoolkit.cistrome.org/).

### Patients

Seventy‐two patients with OSCC who underwent surgical resection at the Fourth Affiliated Hospital of Hebei Medical University from January 2005 to December 2010 were enrolled in this study. All patients were pathologically confirmed with OSCC. OSCC and adjacent normal tissues (> 2 cm from the tumor margin) were collected. None of these patients were receiving chemotherapy, radiotherapy, or other cancer‐related treatment prior to surgery. Written informed consent was obtained from all enrolled patients. The study methodologies conformed to the standards set by the Declaration of Helsinki. This study was approved by the Ethics Committee of the Fourth Affiliated Hospital of Hebei Medical University (Approval No. 2022KY391).

### Prognostic analysis

Gene expression and clinical data of the 72 OSCC patients collected in this study was used for prognostic analysis. Patients were divided into *MEG3*‐high and *MEG3*‐low expression groups according to the median of *MEG3* expression. Overall survival (OS) and progression‐free survival (PFS) were analyzed by Kaplan–Meier plots and log‐rank tests with the r package ‘survival.’ *P* < 0.05 was considered a significant difference.

### Cell culture and treatment

Four OSCC cell lines, SCC4, SCC9, SCC25, and Cal27, and one human normal oral keratinocyte line (NOK), primary gingival keratinocytes, were obtained from the American Type Culture Collection (ATCC, Rockville, MD, USA). Cells were cultured in Dulbecco's modified Eagle medium (DMEM) medium (Gibco, Gaithersburg, MD, USA) containing 10% FBS (Gibco) and 1% penicillin–streptomycin (Gibco) at 37 °C with 5% CO_2_. To investigate whether *MEG3* downregulation in OSCC cells is associated with H3K27me3 modification of the *MEG3* gene locus, Cal27 and SCC4 cells were treated with 5 μm enhancer of zeste homolog 2 (EZH2) inhibitor GSK126 (Beyotime, Shanghai, China) at 37 °C for 36 h.

### Cell transfection

*MEG3* overexpression plasmid (oe‐*MEG3*), empty plasmid (oe‐Control), siRNA targeting GATA3 (si‐GATA3), and siRNA negative control (si‐NC) were purchased from GenePharma (Shanghai, China). Cal27 and SCC4 cells were seeded into 6‐well plates and cultured to 70% confluence followed by cell transfection. Lipofectamine 3000 (Invitrogen, La Jolla, CA, USA) was used for cell transfection according to the manufacturer's instructions. Cells were harvested 48 h after transfection for subsequent analysis.

### Quantitative real‐time polymerase chain reaction (qRT‐PCR)

Total RNA of OSCC cells or tissues was extracted using TRIzol (Invitrogen). RNA was reverse transcribed to cDNA using the PrimeScript RT reagent Kit (TaKaRa, Kyoto, Japan). qRT‐PCR amplification was performed using the SYBR Green qPCR Master Mix Kit (TaKaRa) according to the manufacturer's instructions. GAPDH was employed as the internal reference. Relative gene expression was calculated according to the 2^−ΔΔCt^ method. The sequences of primers were as follows: *MEG3*, forward, 5'‐CTTTTCTGGGGGAATGGGG‐3′; reverse, 5'‐AGAGGGGTGGGAAGGGACT‐3′. HIC1, forward, 5'‐GTCGTGCGACAAGAGCTACAA‐3′; reverse, 5'‐CGTTGCTGTGCGAACTTGC‐3′. PRICKLE1, forward, 5'‐TTTGCTTGCTTACCAGAGGAAA‐3′; reverse, 5'‐ACTGGCAATACCGTACCTCAT‐3′. GATA3, forward, 5'‐GCCCCTCATTAAGCCCAAG‐3′; reverse, 5'‐TTGTGGTGGTCTGACAGTTCG‐3′. GAPDH, forward, 5'‐GGAGCGAGATCCCTCCAAAAT‐3′; reverse, 5'‐GGCTGTTGTCATACTTCTCATGG‐3′.

### Chromatin immunoprecipitation (ChIP)‐qPCR

Cal27 and SCC4 cells in the GSK126 treatment group and control group were cross‐linked with 1% formaldehyde for 10 min and then lysed with cell lysis solution for 20 min. The cross‐linked chromatin was sonicated with Covaris E220 (Woburn, MA, USA). Immunoprecipitation with anti‐H3K27me3 (#ab6002, Abcam, Cambridge, MA, USA) and anti‐IgG (#ab133470, Abcam) was performed overnight at 4 °C. The DNA was purified using a Gel Extraction Kit (Omega Bio‐Tek, Norcross, GA, USA). Finally, qRT‐PCR was performed using the purified products. The sequences of primers were as follows: Region 1, forward, 5'‐CAAGTCCCCGCAGATGAAGT‐3′; reverse, 5'‐CAGGACACAGGGCACCTTAG‐3′. Region 2, forward, 5'‐GTCAAACGCATACCCTCCCA‐3′; reverse, 5'‐GGGTCAGACACCCCAATGAG‐3′.

### RNA immunoprecipitation (RIP)‐qPCR

Cal27 and SCC4 cells were lysed with RIPA lysis buffer (Solarbio, Beijing, China). Binding of YTHDC1 or GATA3 to *MEG3* was detected using anti‐YTHDC1 (#ab264375, Abcam), anti‐GATA3 (#ab199428, Abcam), and anti‐IgG (#ab133470, Abcam) and the Magna RIP Kit (Millipore, Billerica, MA, USA) according to the manufacturer's protocol. The m6A modification of *MEG3* was detected using anti‐m6A (#ab208577, Abcam), anti‐IgG (#ab133470, Abcam), and Magna MeRIP™ m6A Kit (Millipore) according to the manufacturer's instructions. Relative enrichment of RNA was measured by qRT‐PCR. The sequences of primers were as follows: *MEG3*, forward, 5'‐CTTTTCTGGGGGAATGGGG‐3′; reverse, 5'‐AGAGGGGTGGGAAGGGACT‐3′.

### Cell counting kit 8 (CCK8) assay

Cell proliferation was assessed using the CCK8 kit (Solarbio). Cal27 and SCC4 cells were seeded into 96‐well plates at 5 × 10^3^ cells per well, and then incubated at 37 °C with 5% CO_2_. CCK8 solution (10 μL) was added to each well at each timepoint (0, 24, 48, and 72 h) and incubated at 37 °C in the dark for 2 h. The optical density (OD) at 450 nm was measured using a microplate reader (Thermo, Waltham, MA, USA).

### Transwell assay

Cal27 and SCC4 cells were resuspended in serum‐free DMEM medium and adjusted to a concentration of 1.0 × 10^5^ cells·mL^−1^. Transwell chambers were precoated with Matrigel (BD Biosciences, San Jose, CA, USA). Two hundred microliters of cell suspension (with serum‐free DMEM) was added into the apical chamber, and 600 μL of DMEM with 10% fetal bovine serum (FBS) was added into the basolateral chamber. After 48 h of incubation, cells on the upper surface of the chambers were discarded, while cells on the lower surface were fixed with 5% paraformaldehyde for 20 min and stained with 0.1% crystal violet for 20 min.

### Statistical analysis

Statistical analysis was performed using r software 3.6 (https://www.r‐project.org/). Data are shown as mean ± SD. One‐way analysis of variance (ANOVA) followed by Tukey's test and Student's *t*‐test were conducted for comparison of multiple and two groups, respectively. *P* < 0.05 indicated the significant difference.

## Results

### Low expression of *MEG3* was associated with poor prognosis in OSCC patients

**Fig. 1:**
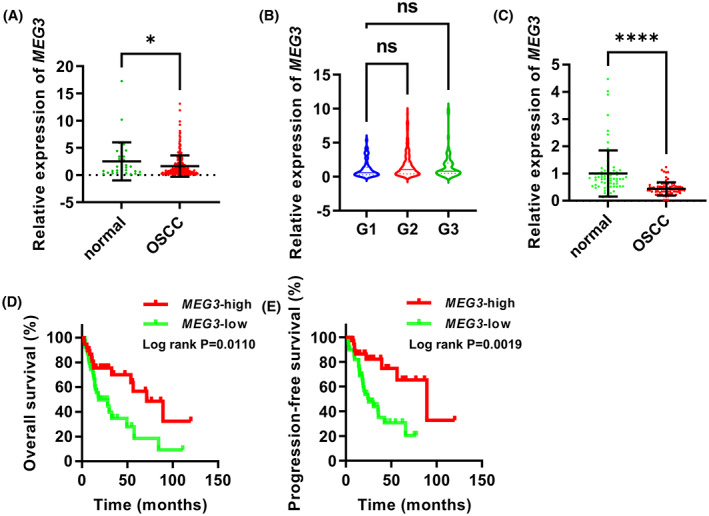
Low expression of *MEG3* was associated with poor prognosis in OSCC patients. (A) Analysis of *MEG3* expression in 324 OSCC tissues and 32 normal control tissues based on TCGA data. Student's *t*‐test was applied for statistical analysis. Data are shown as median ± SD. *P < 0.05. (B) Analysis of the differences in *MEG3* expression among G1, G2, and G3 tumor grades based on TCGA data. ANOVA followed by Tukey's test was performed for statistical analysis. Ns, nonsignificant. Solid line, median. Dotted line, quartile. (C) qRT‐PCR was performed to analyze *MEG3* expression in 72 pairs of OSCC and adjacent normal tissues collected in this study. Student's *t*‐test was applied for statistical analysis. Data are shown as median ± SD. ****P < 0.0001. (D,E) overall survival (D) and progression‐free survival (E) of *MEG3*‐high (*n* = 36) and *MEG3*‐low (*n* = 36) groups based on the 72 patients enrolled in this study. The log‐rank Kaplan–Meier survival test was applied to compare the survival distribution of *MEG3*‐high and *MEG3*‐low groups.

To investigate how *MEG3* affects OSCC progression, we analyzed the expression of *MEG3* in 324 OSCC tissues and 32 normal control tissues based on the TCGA database. The expression of *MEG3* was significantly downregulated in OSCC tissues relative to the normal control tissues, with a 0.4‐fold downregulation (*P* < 0.05; Fig. 1A). However, there was no significant difference in *MEG3* expression among G1, G2, and G3 grades of OSCC (Fig. 1B). Furthermore, the expression of *MEG3* in 72 pairs of OSCC and adjacent normal tissues collected in this study was detected by qRT‐PCR. Compared with the normal control tissues, *MEG3* expression was significantly downregulated in OSCC tissues (0.6‐fold downregulation, *P* < 0.0001; Fig. 1C).

To further assess the impacts of *MEG3* on the prognosis of OSCC patients, 72 OSCC patients enrolled in this study were divided into *MEG3*‐high (*n* = 36) and *MEG3*‐low expression (*n* = 36) groups based on the median of *MEG3* expression. It was found that patients in the *MEG3*‐low group had a worse OS than those in the *MEG3*‐high group (Fig. 1D). In addition, low expression of *MEG3* corresponded to a poor PFS of OSCC patients (Fig. 1E). Hence, we concluded that *MEG3* was downregulated in OSCC tissues, and low expression of *MEG3* was related to poor prognosis in OSCC patients.

### H3K27me3 modification of *MEG3* gene locus resulted in the downregulation of *MEG3* in OSCC cells

**Fig. 2:**
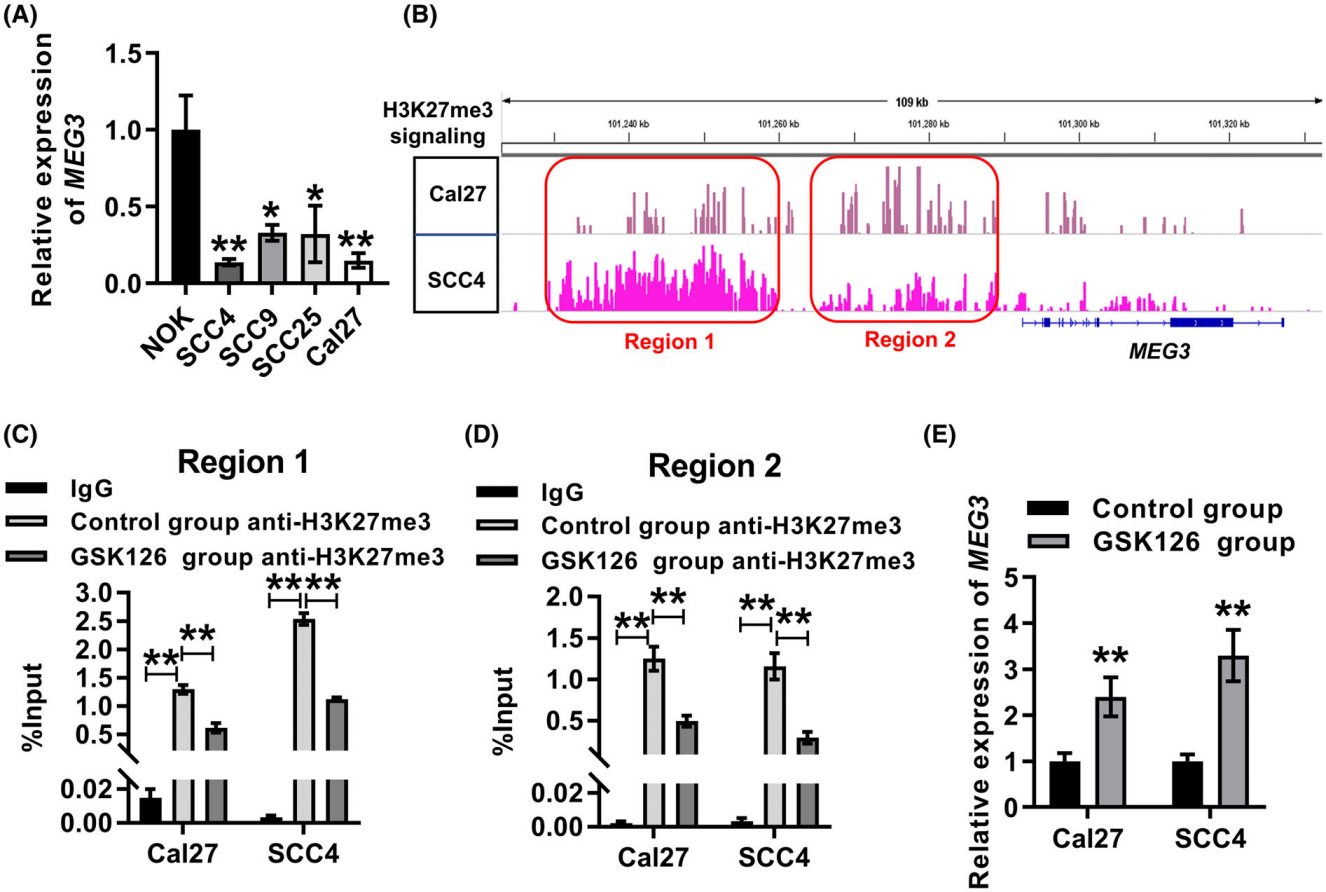
H3K27me3 modification of the *MEG3* gene locus resulted in the downregulation of *MEG3* in OSCC cells. (A) qRT‐PCR was used to measure the relative expression of *MEG3* in SCC4, SCC9, SCC25, Cal27, and NOK cells. ANOVA followed by Tukey's test was performed for statistical analysis. *P < 0.05, **P < 0.01, vs. NOK cells. (B) Enrichment analysis of H3K27me3 signal of *MEG3* gene locus in Cal27 and SCC4 cells based on the GSE149670 dataset. The H3K27me3‐enriched region was divided into region 1 and 2. (C,D) ChIP‐qPCR was used to detect the H3K27me3 modification of region 1 (C) and 2 (D) in Cal27 and SCC4 cells with or without GSK126 treatment. ANOVA followed by Tukey's test was applied for statistical analysis. **P < 0.01. (E) qRT‐PCR was used to measure the relative expression of *MEG3* in Cal27 and SCC4 cells with or without GSK126 treatment. Student's *t*‐test was applied for statistical analysis. **P < 0.01. Experiments were performed in three biologically‐independent replicates. Data shown as mean ± SD.

To further investigate the expression pattern of *MEG3* in OSCC, we examined the expression of *MEG3* in OSCC cells using qRT‐PCR. The results revealed that *MEG3* expression was dramatically downregulated in OSCC cells (SCC4, SCC9, SCC25, and Cal27) relative to NOK cells (Fig. 2A). In the present study we explored the underlying reasons for the low expression of *MEG3* in OSCC cells. EZH2‐catalyzed H3K27me3 is a key factor in the repression of gene transcription [30, 31]. We speculated that the low expression of *MEG3* in OSCC cells was associated with H3K27me3 modification of the *MEG3* gene locus. To confirm this speculation, the H3K27me3 signal of *MEG3* gene locus in two OSCC cells (SCC4 and Cal27 cells) was analyzed according to GSE149670. The results showed that the H3K27me3 signal of the *MEG3* gene locus was remarkably enriched in SCC4 and Cal27 cells (Fig. 2B). We segmented the H3K27me3 signal enrichment region into regions 1 and 2 (Fig. 2B). Subsequently, H3K27me3 modification of regions 1 and 2 was verified using ChIP‐qPCR (Fig. 2C,D). GSK126 is an EZH2 inhibitor that reduces cellular H3K27me3 levels [32]. Cal27 and SCC4 cells were treated with 5 μm GSK126. The results of ChIP‐qPCR suggested that GSK126 treatment significantly downregulated the H3K27me3 modification of regions 1 and 2 in Cal27 and SCC4 cells (Fig. 2C,D). Then, *MEG3* expression in the GSK126 group and the control group were detected by qRT‐PCR. It was found that the expression level of *MEG3* was significantly higher in the GSK126 group than the control group (Fig. 2E). Taken together, these results suggested that *MEG3* was downregulated in OSCC cells, and that this downregulation was associated with H3K27me3 modification on the *MEG3* gene locus.

### Overexpression of *MEG3* inhibited the proliferation and invasion of OSCC cells

**Fig. 3:**
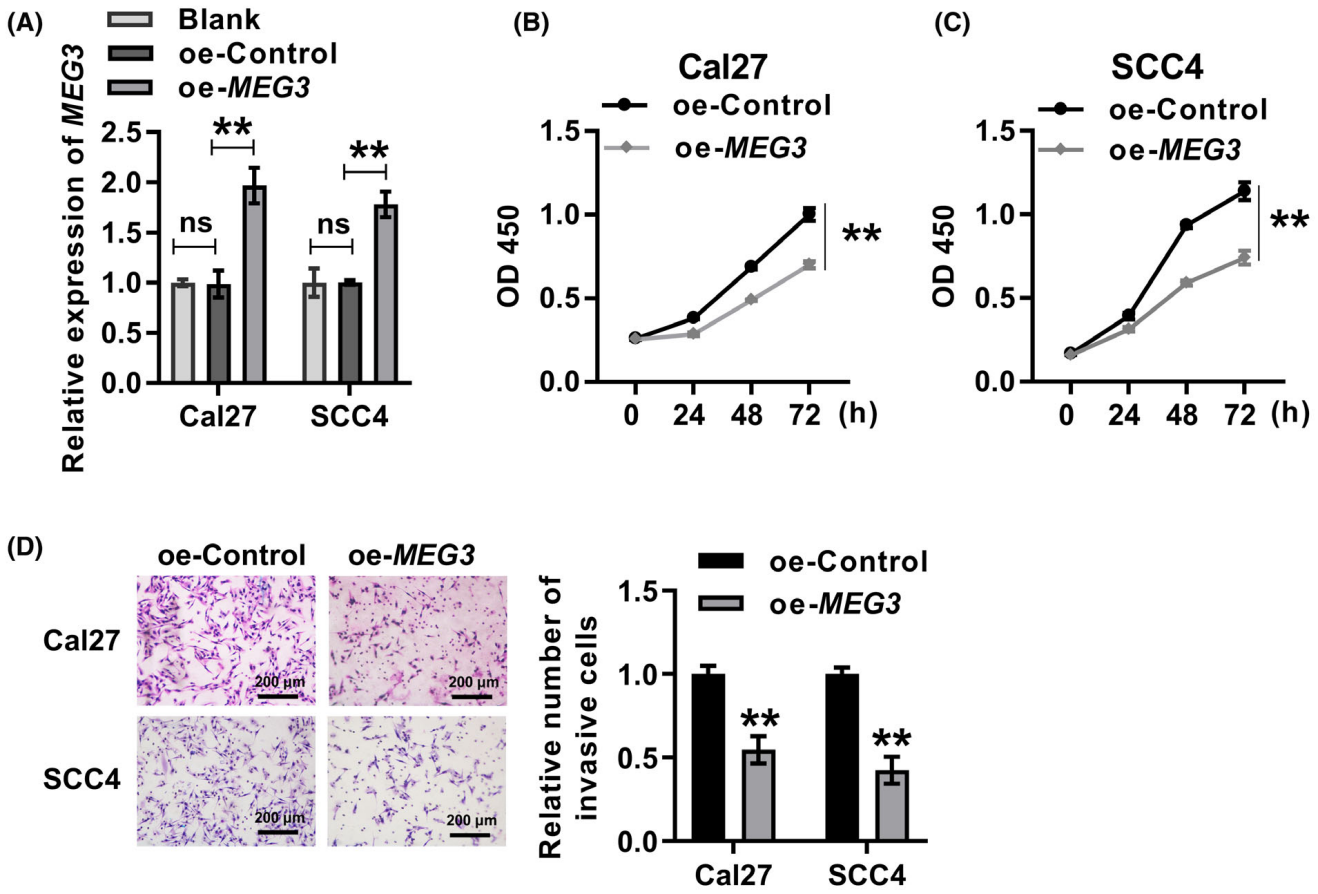
Overexpression of *MEG3* inhibited the proliferation and invasion of OSCC cells. (A) qRT‐PCR was performed to detect the efficiency of *MEG3* overexpression in Cal27 and SCC4 cells. ANOVA followed by Tukey's test was performed for statistical analysis. **P < 0.01. Ns, nonsignificant. (B,C) CCK8 assay was used to detect the proliferation of Cal27 (B) and SCC4 (C) cells in oe‐control and oe‐*MEG3* groups. Student's *t*‐test was applied for statistical analysis. **P < 0.01. (D) Transwell assay was performed to detect the invasion of Cal27 and SCC4 cells in oe‐control and oe‐*MEG3* groups. Student's *t*‐test was applied for statistical analysis. **P < 0.01. Experiments were performed in three biologically‐independent replicates. Data shown as mean ± SD. Scale bars = 200 μm.

To determine the function of *MEG3* in OSCC progression, *MEG3* was overexpressed in Cal27 and SCC4 cells. *MEG3* expression was significantly upregulated in Cal27 and SCC4 cells by transfection with oe‐*MEG3* plasmid, indicating that *MEG3* overexpression cell lines were successfully obtained (Fig. 3A). Overexpression of *MEG3* significantly inhibited the proliferation of Cal27 and SCC4 cells (Fig. 3B,C). In addition, the Transwell assay showed that the invasion of both Cal27 and SCC4 cells was significantly attenuated after overexpression of *MEG3* (Fig. 3D). These results demonstrated the inhibitory effect of *MEG3* on the proliferation and invasion of OSCC cells.

### *MEG3* was modified by m6A and bound to YTHDC1

**Fig. 4:**
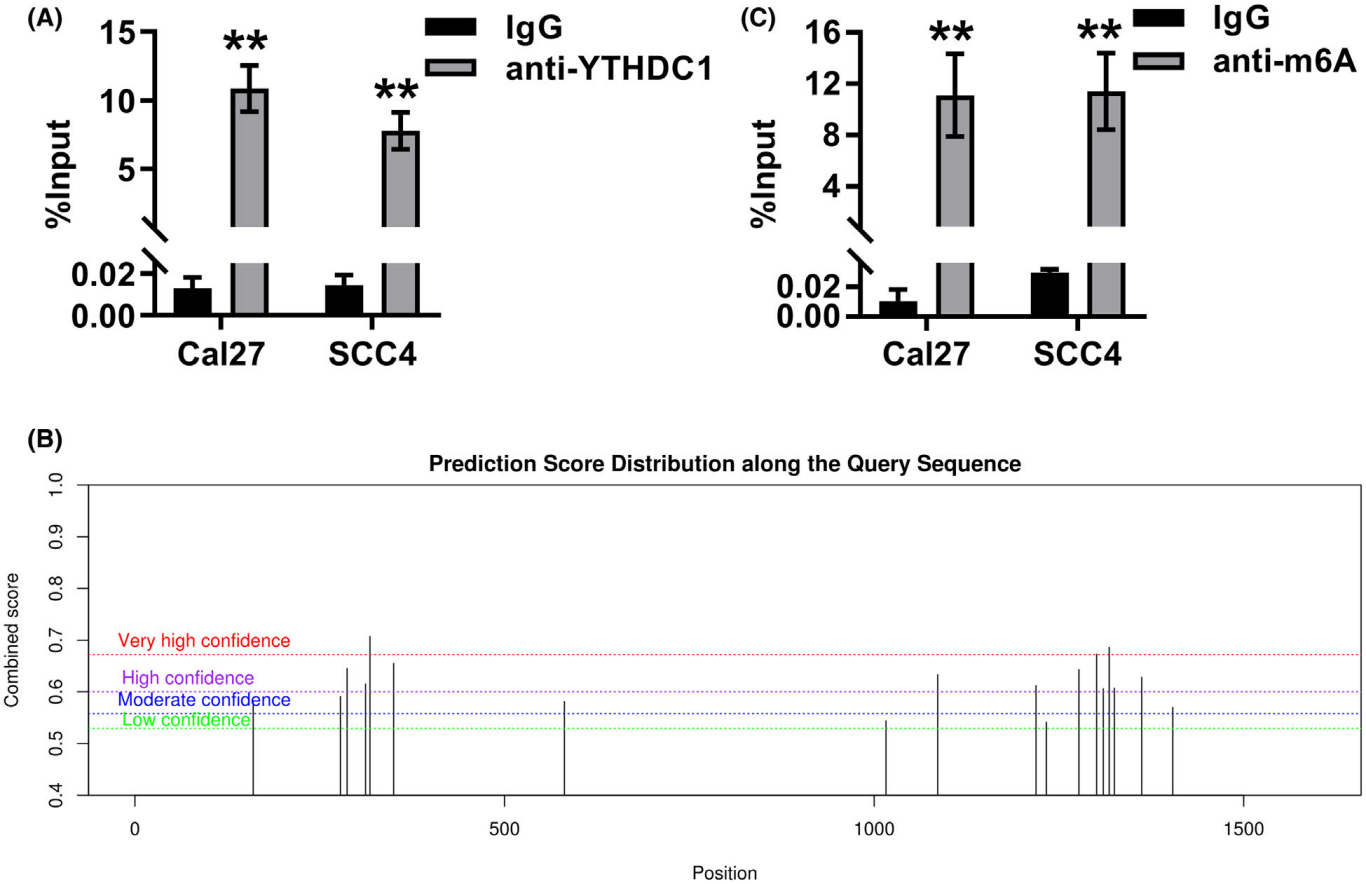
*MEG3* was modified by m6A and bound to YTHDC1. (A) RIP‐qPCR was used to detect the binding between YTHDC1 and *MEG3* in Cal27 and SCC4 cells. Student's *t*‐test was applied for statistical analysis. **P < 0.01. (B) Potential m6A modification sites of *MEG3* were predicted using SRAMP (http://www.cuilab.cn/sramp). (C) RIP‐qPCR was used to detect *MEG3* enriched levels of Cal27 and SCC4 cells in anti‐m6A and IgG groups. Student's *t*‐test was applied for statistical analysis. **P < 0.01. Experiments were performed in three biologically‐independent replicates. Data shown as mean ± SD.

To investigate the mechanism of *MEG3* inhibiting OSCC progression, the binding proteins of *MEG3* were predicted by starbase (https://starbase.sysu.edu.cn/). YTHDC1 was predicted as a potential binding protein of *MEG3* (Table 1). Then, the binding of YTHDC1 to *MEG3* was confirmed by RIP‐qPCR (Fig. 4A). Given that YTHDC1 is an m6A reader [33], SRAMP (http://www.cuilab.cn/sramp/) was used to predict the m6A sites of *MEG3*. The predicted results of SRAMP indicated the presence of abundant m6A sites in *MEG3*, suggesting that *MEG3* may be modified by m6A (Fig. 4B). RIP‐qPCR results showed a significantly higher enrichment of *MEG3* in the anti‐m6A group compared to the IgG group, confirming the presence of m6A modification of *MEG3* (Fig. 4C). Taken together, these results suggested that *MEG3* was modified by m6A and bound to YTHDC1.

**Table 1:** The potential binding proteins of *MEG3*.

Proteins	Official full name	Gene ID
YTHDC1	YTH domain containing 1	ENSG00000083896
XRN2	5′–3′ exoribonuclease 2	ENSG00000088930
UPF1	UPF1 RNA helicase and ATPase	ENSG00000005007
U2AF2	U2 small nuclear RNA auxiliary factor 2	ENSG00000063244
U2AF1	U2 small nuclear RNA auxiliary factor 1	ENSG00000160201
TRA2A	Transformer 2 alpha homolog	ENSG00000164548
TNRC6A	Trinucleotide repeat containing adaptor 6A	ENSG00000090905
TARDBP	TAR DNA binding protein	ENSG00000120948
TAF15	TATA‐box binding protein associated factor 15	ENSG00000270647
SRSF3	Serine and arginine rich splicing factor 3	ENSG00000112081
SRSF1	Serine and arginine rich splicing factor 1	ENSG00000136450
SND1	Staphylococcal nuclease and tudor domain containing 1	ENSG00000197157
SMNDC1	Survival motor neuron domain containing 1	ENSG00000119953
SAFB2	Scaffold attachment factor B2	ENSG00000130254
RBM6	RNA binding motif protein 6	ENSG00000004534
RBM5	RNA binding motif protein 5	ENSG00000003756
RBM10	RNA binding motif protein 10	ENSG00000182872
QKI	QKI, KH domain containing RNA binding	ENSG00000112531
PTBP1	Polypyrimidine tract binding protein 1	ENSG00000011304
PRPF8	pre‐mRNA processing factor 8	ENSG00000174231
NOP58	NOP58 ribonucleoprotein	ENSG00000055044
NOP56	NOP56 ribonucleoprotein	ENSG00000101361
MSI1	Musashi RNA binding protein 1	ENSG00000135097
MBNL2	Muscleblind like splicing regulator 2	ENSG00000139793
LSM11	LSM11, U7 small nuclear RNA associated	ENSG00000155858
KHSRP	KH‐type splicing regulatory protein	ENSG00000088247
KHDRBS1	KH RNA binding domain containing, signal transduction associated 1	ENSG00000121774
ILF3	Interleukin enhancer binding factor 3	ENSG00000129351
IGF2BP2	Insulin like growth factor 2 mRNA binding protein 2	ENSG00000073792
HNRNPUL1	Heterogeneous nuclear ribonucleoprotein U like 1	ENSG00000105323
HNRNPU	Heterogeneous nuclear ribonucleoprotein U	ENSG00000153187
HNRNPK	Heterogeneous nuclear ribonucleoprotein K	ENSG00000165119
HNRNPC	Heterogeneous nuclear ribonucleoprotein C	ENSG00000092199
HNRNPA2B1	Heterogeneous nuclear ribonucleoprotein A2/B1	ENSG00000122566
HNRNPA1	Heterogeneous nuclear ribonucleoprotein A1	ENSG00000135486
FUS	FUS RNA binding protein	ENSG00000089280
FMR1	Fragile X messenger ribonucleoprotein 1	ENSG00000102081
FBL	Fibrillarin	ENSG00000105202
EWSR1	EWS RNA binding protein 1	ENSG00000182944
ELAVL1	ELAV like RNA binding protein 1	ENSG00000066044
EIF4G2	Eukaryotic translation initiation factor 4 gamma 2	ENSG00000110321
EIF4A3	Eukaryotic translation initiation factor 4A3	ENSG00000141543
DKC1	Dyskerin pseudouridine synthase 1	ENSG00000130826
DGCR8	DGCR8 microprocessor complex subunit	ENSG00000128191
CSTF2T	Cleavage stimulation factor subunit 2 tau variant	ENSG00000177613
CPSF6	Cleavage and polyadenylation specific factor 6	ENSG00000111605
ADAR	Adenosine deaminase RNA specific	ENSG00000160710

### Screening and functional analysis of enhancer‐controlled genes positively regulated by *MEG3*

**Fig. 5:**
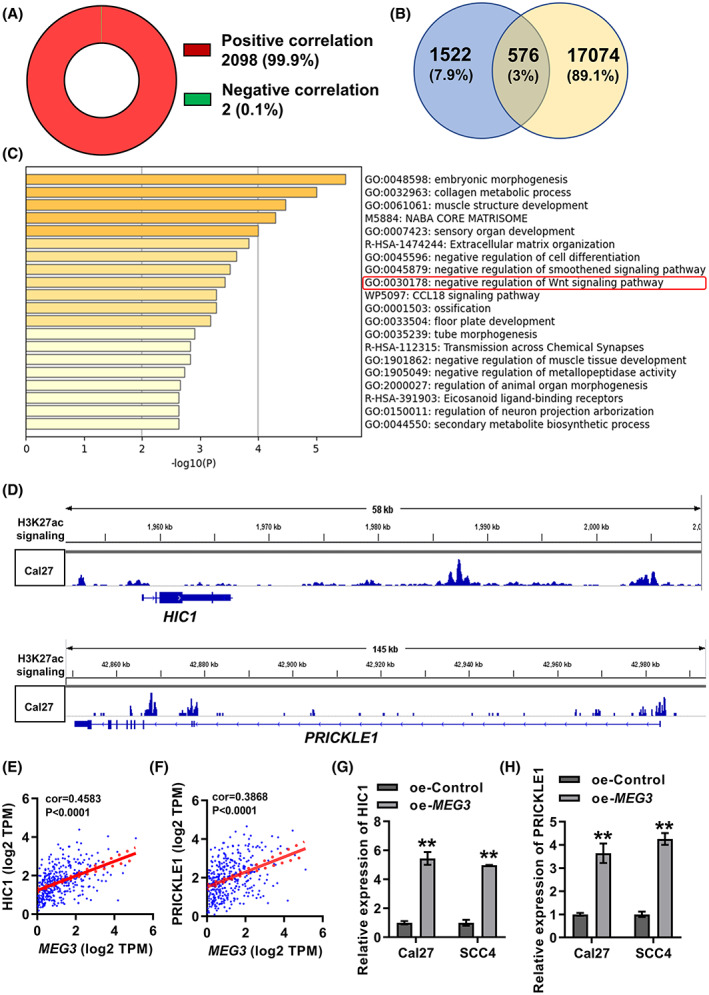
Screening and functional analysis of enhancer‐controlled genes positively regulated by *MEG3*. (A) Genes significantly correlated with *MEG3* expression in OSCC tissues were screened based on TCGA data. (B) Intersections of genes positively correlated with *MEG3* expression (blue, based on TCGA data) and enhancer‐controlled genes (yellow, based on GSE149670). (C) metascape (https://metascape.org/) analysis of the 576 intersecting genes. (D) H3K27ac signal at *HIC1* and *PRICKLE1* gene locus in Cal27 cells based on the GSE149670 dataset. (E,F) Pearson's correlation among *MEG3*, HIC1 (E) and PRICKLE1 (F) transcription in OSCC tissues was assessed based on TCGA data. (G,H) qRT‐PCR was performed to detect the relative transcriptional levels of HIC1 (G) and PRICKLE1 (H) in *MEG3* overexpressed and control cells. Student's *t*‐test was applied for statistical analysis. **P < 0.01. Experiments were performed in three biologically‐independent replicates. Data shown as mean ± SD.

YTHDC1 interacts with m6A‐modified RNA and bromodomain containing 4 (BRD4) to induce the recruitment of transcription factors at the target gene promoter, thereby activating the transcription of target gene [33, 34, 35]. BRD4 recognizes H3K27ac, an epistatic marker for activated enhancers, which is required for gene transcriptional activation [36]. Therefore, we hypothesized that *MEG3* triggers the transcription of target genes through regulating their enhancers. Based on data from TCGA, we screened genes significantly associated with *MEG3* expression in OSCC tissues, resulting in 2098 positively and 2 negatively genes associated with *MEG3* expression (Fig. 5A). Based on the GSE149670 dataset, 17 650 gene loci rich in H3K27ac signal were found in Cal27 cells, and these genes were identified as enhancer‐controlled genes. Then, a total of 576 intersecting genes were filtered out by screening the intersections of genes positively correlated with *MEG3* expression (*n* = 2098) and enhancer‐controlled genes (*n* = 17 650; Fig. 5B). These 576 genes were defined as enhancer‐controlled genes positively regulated by *MEG3*.

Functional analysis of the 576 intersecting genes by the metascape platform revealed that these genes were significantly enriched in ‘negative regulation of Wnt signaling pathway’ (Fig. 5C). A total of 20 genes were enriched in ‘negative regulation of Wnt signaling pathway,’ and two of them, *HIC1* and *PRICKLE1*, were randomly selected for validation. Figure 5D details the modification of *HIC1* and *PRICKLE1* gene loci by H3K27ac. Based on TCGA data, *MEG3* showed a significant positive correlation with the transcription of HIC1 and PRICKLE1 (Fig. 5E,F). Furthermore, the transcriptional levels of HIC1 and PRICKLE1 in Cal27 and SCC4 cells overexpressing *MEG3* were detected by qRT‐PCR. The results showed that the overexpression of *MEG3* significantly promoted the transcription of HIC1 and PRICKLE1 (Fig. 5G,H). Overall, we screened 576 enhancer‐controlled genes positively regulated by *MEG3*, and found that these genes were associated with ‘negative regulation of Wnt signaling pathway’.

### The anticancer function of *MEG3* in OSCC was dependent on the interaction with GATA3

**Fig. 6:**
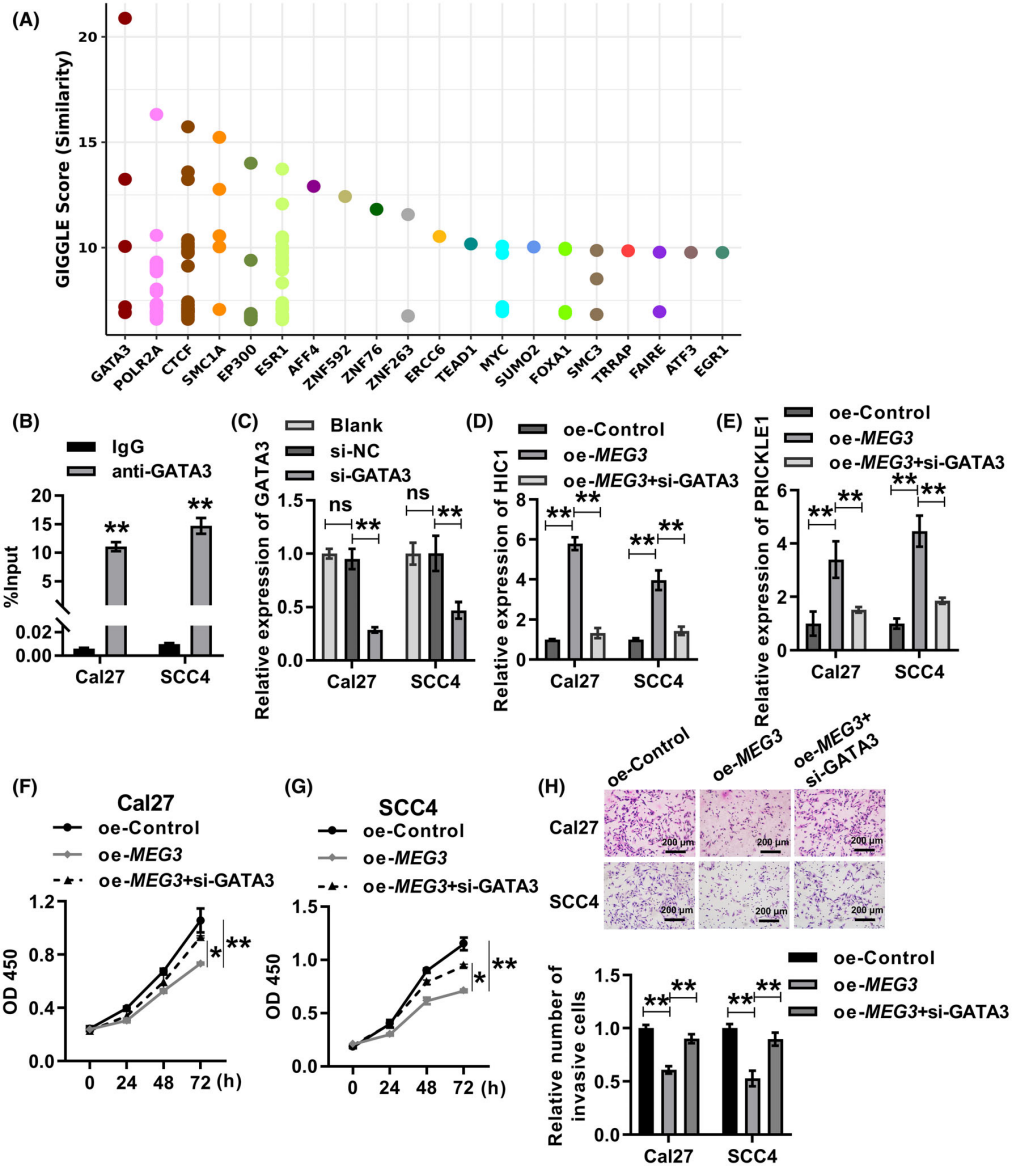
The anticancer function of *MEG3* in OSCC was dependent on the interaction with GATA3. (A) Transcription factors of the 576 intersecting genes were predicted using the toolkit for the cistrome data browser (http://dbtoolkit.cistrome.org/). (B) RIP‐qPCR was used to measure the binding between GATA3 and *MEG3* in Cal27 and SCC4 cells. Student's *t*‐test was applied for statistical analysis. **P < 0.01. (C) Knockdown efficiency of GATA3 in Cal27 and SCC4 cells was assessed by qRT‐PCR. ANOVA followed by Tukey's test was performed for statistical analysis. **P < 0.01. Ns, nonsignificant. (D,E) qRT‐PCR was used to assess the relative transcription levels of HIC1 (D) and PRICKLE1 (E) in Cal27 and SCC4 cells after overexpression of *MEG3* alone or overexpression of *MEG3* with concomitant knockdown of GATA3. ANOVA followed by Tukey's test was performed for statistical analysis. **P < 0.01. (F,G) CCK8 assay was performed to detect the proliferation of Cal27 (F) and SCC4 (G) cells after overexpression of *MEG3* alone or overexpression of *MEG3* with concomitant knockdown of GATA3. ANOVA followed by Tukey's test was performed for statistical analysis. *P < 0.05, **P < 0.01. (H) Transwell assay was performed to detect invasion of Cal27 and SCC4 cells after overexpression of *MEG3* alone or overexpression of *MEG3* with concomitant knockdown of GATA3. ANOVA followed by Tukey's test was performed for statistical analysis. **P < 0.01. Experiments were performed in three biologically‐independent replicates. Data shown as mean ± SD. Scale bars = 200 μm.

In order to develop more detailed understanding of the mechanisms by which *MEG3* inhibits OSCC progression, we wished to identify transcription factors involved in regulating of enhancer‐controlled genes positively regulated by *MEG3*. Transcription factors of the 576 intersecting genes were predicted using the Toolkit for cistrome data Browser (http://dbtoolkit.cistrome.org/). The top 20 transcription factors, with GATA3 ranked first, are shown in Fig. 6A. The RIP‐qPCR results showed that *MEG3* was significantly more stronger enriched in anti‐GATA3 group than in the IgG group, which demonstrated the binding between *MEG3* and GATA3 (Fig. 6B). Cal27 and SCC4 cells with GATA3 knockdown were successfully established by transfecting si‐GATA3 (Fig. 6C). Overexpression of *MEG3* significantly promoted the transcription of HIC1 and PRICKLE1, which was restored by knocking down GATA3 (Fig. 6D,E). The CCK8 assay revealed that overexpression of *MEG3* inhibited the proliferation of Cal27 and SCC4 cells, while knockdown of GATA3 partially counteracted this inhibitory effect (Fig. 6F,G). The invasion of Cal27 and SCC4 cells was inhibited after overexpression of *MEG3*, and this inhibition was reversed by GATA3 knockdown (Fig. 6H). All of these results indicated that the anticancer function of *MEG3* in OSCC was dependent on the interaction with GATA3.

## Discussion

Oral squamous cell carcinoma is a common malignant tumor of the head and neck [1, 2, 5]. Mechanisms underlying the development of OSCC are unclear. Aberrant expression of lncRNAs plays important roles in regulating tumor progression [37, 38]. This study confirmed the anticancer function of *MEG3* in OSCC progression. We found that downregulation of *MEG3* in OSCC cells was associated with H3K27me3 modification of the *MEG3* gene locus. We also provided evidence that *MEG3* was bound to YTHDC1 and modified by m6A in OSCC cells. In addition, the anticancer function of *MEG3* was relying on binding to the transcription factor GATA3.

*MEG3* is usually acts as a tumor‐suppressive lncRNA, which is downregulated in tumor tissues and cells [9, 10, 11, 12, 13]. Several studies have shown that *MEG3* expression is inhibited in OSCC tissues [14, 15, 16]. Consistent with this, we found that *MEG3* expression was downregulated in OSCC tissues by analyzing TCGA data and the 72 pairs samples collected in this study. However, the prognostic value of *MEG3* in OSCC patients has not been clarified. In the present study we found that, although the differences in *MEG3* expression among G1, G2, and G3 grades were not significant, patients with low *MEG3* expression had a worse outcome.

The underlying mechanism for the downregulation of *MEG3* in OSCC cells is unclear. As we know, EZH2 could be recruited to target genes, and subsequently catalyzes the 27th lysine trimethylation of nucleosome histone H3, which is one of the central mechanisms of gene expression repression [39]. In this study it was confirmed that there was abundant H3K27me3 signal of the *MEG3* gene locus in OSCC cells. GSK126 is a highly selective S‐adenosyl‐methionine‐competitive EZH2 inhibitor that reduces cellular H3K27me3 levels [32]. We found that GSK126 treatment indeed significantly inhibited H3K27me3 modification of the *MEG3* gene locus and promoted *MEG3* expression in OSCC cells, suggesting that the downregulation of *MEG3* expression in OSCC cells was mediated by H3K27me3 modification of the *MEG3* gene locus.

Several reports have demonstrated that *MEG3* inhibits proliferation, migration, invasion, and promotes apoptosis of OSCC cells [14, 15, 16]. In the present work, it was authenticated that overexpression of *MEG3* resulted in the suppression of proliferation and invasion of OSCC cells, which is consistent with previous studies [14, 15, 16]. Regarding the regulatory mechanism of *MEG3*, previous studies have found that *MEG3* inhibits the malignant phenotype of OSCC cells by targeting miR‐548d‐3p and miR‐21 [14, 15]. Additionally, *MEG3* promotes p53 expression and directly binds to p53, thereby activating the p53 signaling pathway and suppressing the malignant phenotype of tumor cells [16, 40, 41]. However, the detailed mechanism by which *MEG3* inhibits OSCC remains poorly understood. This study demonstrated the binding between *MEG3* and YTH domain containing 1 (YTHDC1). YTHDC1 is a nuclear m6A reader [33]. m6A is the most abundant posttranscriptional base modification found in eukaryotic RNA [42]. m6A‐modified lncRNAs can be recognized and bound by YTHDC1, which in turn affects cellular biological processes [43, 44]. However, we did not find any studies of m6A‐modified *MEG3* in OSCC cells. In this study, we found that *MEG3* was modified by m6A in OSCC cells. These results inspired us to explore the anticancer mechanism of *MEG3* from the perspective of YTHDC1 regulation.

YTHDC1 interacts with m6A‐modified RNAs and BRD4, to promote the recruitment of transcription factors and the activation of enhancers [33, 34, 35]. According to TCGA data, genes significantly correlated with *MEG3* expression in OSCC tissues were identified. In addition, 17 650 enhancer‐controlled genes in Cal27 cells were filtered. Through intersection analysis, 576 enhancer‐controlled genes positively regulated by *MEG3* were screened. Functional analysis of the 576 genes suggested that these genes were significantly enriched in ‘negative regulation of Wnt signaling pathway.’ As a prior study showed, *MEG3* exerts tumor‐suppressive effects in OSCC by inhibiting of the Wnt/β‐catenin signaling pathway, which is consistent with our results [23]. Furthermore, *HIC1* and *PRICKLE1*, which were enriched in ‘negative regulation of Wnt signaling pathway,’ were selected for validation. HIC ZBTB transcriptional repressor 1 (HIC1) usually plays a suppressive role in tumor progression [45]. Prickle planar cell polarity protein 1 (PRICKLE1) is a negative regulator of the Wnt/beta‐catenin signaling pathway [46]. We found that *HIC1* and *PRICKLE1* gene loci were modified by H3K27ac, suggesting that these two genes were enhancer‐controlled genes. Transcription of these two genes were positively correlated with *MEG3* expression in OSCC tissues. HIC1 and PRICKLE1 transcription were significantly enhanced after overexpression of *MEG3* in OSCC cells.

To further elucidate the tumor‐suppressive mechanism of *MEG3*, we predicted transcription factors of the intersecting genes, and confirmed the binding between *MEG3* and transcription factor GATA3 in OSCC cells. GATA binding protein 3 (GATA3) is a highly conserved transcription factor that plays a pro‐ or anticancer role in cancer progression [47, 48]. For example, GATA3 acts as a downstream gene of BRCA1 to inhibit epithelial mesenchymal transition in breast cancer cells [48]. GATA3 expression promotes proliferation and migration in high‐grade serous ovarian cancer, and is associated with a poor prognosis of high‐grade serous ovarian cancer [49]. However, the function and mechanism of GATA3 in the development of OSCC remain largely unknown. We found that knockdown of GATA3 restored the elevated transcription of HIC1 and PRICKLE1 induced by *MEG3* overexpression. Functional experiments showed that the proliferation and invasion of OSCC cells were reduced after overexpression of *MEG3*, while this reduction was attenuated by GATA3 knockdown.

## Conclusion

In summary, this study confirmed that *MEG3* was downregulated in OSCC cells, which was mediated by H3K27me3 modification of the *MEG3* gene locus. Furthermore, the anticancer function of *MEG3* in OSCC cells was dependent on the interaction with GATA3. These findings have the potential to provide a basis for the discovery of novel therapeutic targets for OSCC.

## Conflict of interest

The authors declare no conflict of interest.

## Author contributions

YQ, LJ, YH and FL conceived this study. YH and FL performed data analysis. YH, FL, NL, XY, LZ and SZ participated in the experiments of this study. YH and FL contributed to the writing of this article. The article was approved by all the authors.

## Data Availability

The presented data can be obtained under reasonable request by contacting Yongle Qiu.
